# Causes and Treatment of Sterility in Both Sexes, and Fecundation by Artificial Methods

**Published:** 1891-02

**Authors:** 


CAUSES AND TREATMENT OF STERILITY IN BOTH SEXES, AND FECUNDATION BY ARTIFICIAL METHODS. Translated from the French of Dr. J. GERARD, Paris, with notes by Chas. Everett Warren, M. D., Boston, Mass., U. S. A., with 200 illustrations, designed by José Roy. Printed for *private* use only by the profession. Boston, Mass.: Published by the *International Medical Exchange.*

This is the title of a very curious book in six parts, the first of which only is before us. The publishers say in their introduction: "This unique work, produced in miniature from the French, nominally relates to sterility, but incidentally introduces, in an amusing colloquial manner, many facts relating to the hygiene of the sexual organs and the relations of the sexes, etc."

A first glance at this remarkable production is hardly calculated to recommend it to the earnest student of the mysteries of which it treats, but a nearer view will soon convince him that under a pleasant colloquial form the learned author has managed to give a good deal of light upon a very dark subject. This first part treats of latent sterility, and every line gives evidence of consummate knowledge obtained by careful study.

There is, in this first part, nothing new to the well-educated physician, and, indeed, the author takes this for granted by avoiding needless explanations, and thus enabling him to give the greater value to the naked facts in the scientific and natural grouping intended to support his conclusions. The humorous style and fanciful imagery with which he illustrates one of the most important subjects in nature's laboratory, while it takes nothing from her dignity and is well calculated to excite serious thoughts of a highly practical turn, makes the work amusing as well as instructive.

We wait with impatience the appearance of the whole work which, judging by the specimen, will, no doubt, be a valuable addition to our medical literature.

The illustrations are unique, clever and spirited ; but, though their boldness may serve to call attention and even aid the memory, we think science would be better served by modifying some and omitting others altogether.

